# Obesity induced gut dysbiosis contributes to disease severity in an animal model of multiple sclerosis

**DOI:** 10.3389/fimmu.2022.966417

**Published:** 2022-09-09

**Authors:** Shailesh K. Shahi, Sudeep Ghimire, Peter Lehman, Ashutosh K. Mangalam

**Affiliations:** ^1^ Department of Pathology, University of Iowa, Iowa City, IA, United States; ^2^ Graduate Program in Immunology, University of Iowa, Iowa City, IA, United States; ^3^ Graduate Program in Molecular Medicine, University of Iowa, Iowa City, IA, United States

**Keywords:** obesity, multiple sclerosis, gut microbiota, experimental autoimmune encephalomyelitis, HLA-class II transgenic mice, gut permeability

## Abstract

**Background:**

Multiple sclerosis (MS) is an inflammatory and demyelinating disease of the CNS. The etiology of MS is complex, and results from the interaction of multiple environmental and genetic factors. Although human leukocyte antigen-HLA alleles such as HLA-DR2 and –DR3 are considered the strongest genetic factors, the environmental factors responsible for disease predisposition are not well understood. Recently, diet and gut microbiota have emerged as an important environmental factors linked to the increased incidence of MS. Especially, western diets rich in** **protein and fat have been linked to the increased incidence of obesity. Numerous clinical data indicate a role of obesity and gut microbiota in MS; however, the mechanistic link between gut microbiota and obesity in the pathobiology of MS remains unclear. The present study determines the mechanisms driving MS severity in the context of obesity utilizing a high-fat diet (HFD) induced obese HLA-DR3 class-II transgenic mouse model of MS.

**Methods:**

HLA-DR3 transgenic mice were kept on a standard HFD diet or Normal Chow (NC) for eight weeks. Gut microbiota composition and functional analysis were performed from the fecal DNA of mice. Experimental autoimmune encephalomyelitis-EAE (an animal model of MS) was induced by immunization with the proteolipid protein-PLP_91-110_ peptide in complete Freud’s Adjuvant (CFA) and pertussis toxin.

**Results:**

We observed that HFD-induced obesity caused gut dysbiosis and severe disease compared to mice on NC. Amelioration of disease severity in mice depleted of gut microbiota suggested an important role of gut bacteria in severe EAE in obese mice. Fecal microbiota analysis in HFD mice shows gut microbiota alterations with an increase in the abundance of *Proteobacteria* and *Desulfovibrionaceae* bacteria and modulation of various bacterial metabolic pathways including bacterial hydrogen sulfide biosynthetic pathways. Finally, mice on HFD showed increased gut permeability and systemic inflammation suggesting a role gut barrier modulation in obesity induced disease severity.

**Conclusions:**

This study provides evidence for the involvement of the gut microbiome and associated metabolic pathways plus gut permeability in obesity-induced modulation of EAE disease severity. A better understanding of the same will be helpful to identify novel therapeutic targets to reduce disease severity in obese MS patients.

## Introduction

Multiple sclerosis (MS) is a heterogeneous immune-mediated inflammatory and demyelinating disease of the central nervous system (CNS). MS affects over 2.8 million people worldwide with nearly one million in the US (1, 2). The etiology of MS is complex and results from the interaction between genetic and multiple environmental factors. Genetic factors account for approximately 30% of disease risk as determined from studies of identical twins (3), and among genetic factors, human leukocyte antigen (HLA) genes on chromosome 6 especially certain HLA-class II alleles such as HLA-DR2 and –DR3 are recognized as the strongest genetic factors associated with predisposition to MS (4). In addition, environmental factors account for 70% of disease risk (5), however, how these are linked with the predisposition to, or protection from, MS is unknown. Recently, gut microbiota have emerged as a potential environmental factor that can contribute to the etiopathogenesis of MS (6–10). Additionally, diet is recognized as one of the major factors that contribute to the composition of gut microbiota (11).Western diet, rich in protein and fat have emerged as important factors contributing to the increased incidence of obesity (650 million people worldwide and 100 million in USA) and inflammatory diseases, including MS in developed countries (12, 13). Multiple studies have shown that MS patients have an altered gut microbiota (dysbiosis) compared to healthy individuals (6, 7, 10), and gut microbial dysbiosis has also been linked with obesity (14, 15). However, the mechanism by which interaction between High Fat Diet (HFD) and gut microbiota regulate disease severity/progression in MS is unknow.

Gut microbiota is key to host physiology and energy balance. The transfer of gut microbiota from obese human/mouse donors into germ-free recipient mice have resulted in transfer of obesity indicating the importance of gut microbiota in induction of obesity (16). Additionally, transfer of gut bacteria from MS patients to germ-free mice resulted in severe experimental autoimmune encephalomyelitis-EAE (an animal model of MS) compared with EAE in mice that received microbiota from healthy controls (17), demonstrating that physiology of recipient mice was modified to resemble that of the donors. Furthermore, MS patients with a higher body mass index (BMI) have more severe disease compared to MS patients with low BMI (10). However, to our knowledge, there is no study on the effects of obesity on the gut microbiome and its implications on the disease severity in MS or EAE. Thus, there is a critical need to understand how obesity affects the gut microbiome and the severity of MS/EAE.

Therefore, in the present study, we utilized HFD induced obesity model in HLA-DR3 transgenic mice to show that obese mice develop severe EAE with increased mortality compared to mice on a normal chow (NC) diet. Altered gut microbiota in mice on a HFD group suggest a role of gut microbiome in the development of sever disease in obese mice. Depletion of gut microbiota reduced disease severity in HFD-induced obese mice, suggesting a critical role of gut bacteria in the induction of severe EAE. Altered gut microbiota in HFD-induce obese mice showed an increased abundance of *Proteobacteria* and *Desulfovibrionaceae* family with enrichment of sulfur metabolism, lipopolysaccharide biosynthesis, and long chain fatty acid biosynthesis (LCFA) pathways linked with inflammation. Thus, our study shows that obesity leads to severe EAE disease in HLA-DR3 transgenic mice through alteration of gut microbiota and enrichment of metabolic pathways linked with the induction of pro-inflammatory pathways.

## Materials and methods

### Mice and dietary treatment

HLA-DR3 (lacking endogenous murine major histocompatibility complex (MHC) class II gene express HLA-DRB1*0301) transgenic mice on B6/129 background have been described previously (18). These mice will be referred to as HLA-DR3 transgenic mice throughout the text. 6-8 weeks old mice were placed on either HFD (45 kcal % fat) or Normal chow *ad libitum* for 8 weeks. HFD contained approximately 45 kcal % fat and dextrose were purchased from Research Diets Inc. (D16020603, New Brunswick, New Jersey, USA). Mice in the control group were fed a Normal Chow (NC) diet available at the animal facilities at the University of Iowa (Envigo 7013). Mice were bred and maintained in the University of Iowa animal facility in accordance with NIH and institutional guidelines. All experiments were approved by the Institutional Animal Care and Use Committee at the University of Iowa.

### EAE disease induction and evaluation

HLA-DR3 transgenic mice (8 to 12 weeks old) were immunized subcutaneously in both flanks using 25 µg of PLP_91–110_ peptides (GenScript, NJ, USA) that was emulsified in CFA containing *Mycobacterium tuberculosis* H37Ra (100 μg/mouse; Becton, Dickinson and Company, Sparks, MD, USA) (18). C57BL/6J mice were immunized subcutaneously (s.c.) on day 0 on the both flank with 100 µg of MOG_35-55_ emulsified in CFA containing *Mycobacterium tuberculosis* H37Ra (100 μg/mouse). Pertussis toxin (PTX) (Sigma Chemicals, St. Louis, MO, 80 ng) was administered intraperitoneally (*i.p*.) in both HLA-DR3 (PLP_91–110_ model) and B6 (MOG_35-55_) mice at days 0 and 2 post immunization. Animals were observed daily for clinical disease up to day 25. Disease severity was scored according to the standard 0-5 scoring system described previously (18). Briefly, 0 score for normal; 1, loss of tail tonicity; 2, hind limb weakness; 3, hind limb paralysis; 4, complete hind limb paralysis and forelimb paralysis or weakness; 5, moribund/death.

### Gut microbiota depletion

Gut microbiota was depleted in HLA-DR3 transgenic and C57BL/6J mice kept on HFD or NC diet using antibiotic cocktail (0.5 g/L vancomycin, 1 g//L neomycin, 1 g/L metronidazole, and 1 g/L ampicillin) in drinking water for four weeks as per stander protocol (19). HLA-DR3 transgenic mice or C57BL/6J mice were divided in four groups *viz*. HFD plus antibiotics, NC plus antibiotics, HFD plus water, and NC plus water. HFD plus antibiotics, NC plus antibiotics fed with HFD or NC diet and broad spectrum antibiotic cocktail were supplemented in drinking water for four weeks. Mice in control group (HFD plus water, and NC plus water) were kept on HFD or NC diet and supplemented with water for four weeks. After four weeks on sterile or antibiotic water, EAE were induced disease were recorded in above four groups of mice.

### Microbiome analysis

HLA-DR3 transgenic mice were kept on a HFD and NC and after 8 weeks on diet specific fecal samples were collected. Microbial DNA extraction, 16S rRNA amplicon, and sequencing were performed as described previously (20). Briefly, total DNA was isolated from the samples using Qiagen PowerSoil DNA isolation kit (MoBio now part of QIAGEN, Valencia, CA, USA) using manufacturer’s instructions. The DNA was quantified using Qubit dsDNA HS (High Sensitivity) assay kit (Thermo Fisher Scientific, CA, USA) and kept at -80^0^C until further analysis. Sequencing was performed using MiSeq platform by amplifying V3-V4 region of the 16S rRNA gene. Downstream analysis of *fastq* files was conducted using *dada2* script in R (20, 21), to generate amplicon sequence variants (ASVs) which were then assigned to taxonomy using a naive Bayesian classifier with the Silva database (21). The ASV table was further analyzed in R v4.1.0 using *phyloseq* package (22). Nineteen samples (Pre-diet = 9, HFD=5 and NC=5) were processed to determine the diversity and community composition after removing a sample from prediet group (PreHFD5) which had a low number of reads. Alpha diversity was measured using number of observed ASVs, and Shannon diversity. Kruskal Wallis test followed by pairwise Wilcoxon test was performed and p-value was adjusted using Benjamini Hochberg (BH) method with adjusted p-value cutoff at 0.05. The ASVs were then filtered to remove those whose total abundance across all samples was < 10 reads in at least 20% of the samples. Samples were rarefied to 13,000 reads and beta diversity was analyzed using bray-curtis dissimilarity and weighted unifrac metrices. Furthermore, *picrust2* (23) was used with the ASV table to obtain the functional pathways associated by mapping to MetaCyc database. Linear discrimination of effect size (Lefse) analysis (24) was used to identify the differentially abundant taxa (*lda_cutoff* = 2) and functional pathways (*lda_cutoff* = 3) at *kw_cutoff* = 0.01 *and wilcoxon_cutoff* =0.01.

### Intestinal permeability and inflammatory mediators measurement

To measure intestinal permeability, mice were orally gavaged with 4 kDa FITC labeled dextran and serum was collected at subsequent time points for measurement of fluorescence and compared to pre-oral gavage serum as described previously (25). Fatty Acid Binding Protein 2 (FABP2) concentrations in serum were analyzed by enzyme linked immunosorbent assay (ELISA DuoSet, R&D, MN, USA) according to the manufacturer’s protocol. Concentrations of MCP-1a, CXCL-5, and CCL-11 were measured using cytometric bead array (CBA) kits (BD Bioscience) according to manufacturer’s instructions.

### Statistical analysis

When comparing average clinical EAE scores, differences between groups were assessed by 2-way ANOVA Sidak multiple comparisons test. Mann–Whitney rank-sum test when comparing only two groups in cumulative EAE scores. Statistical analyses were done with GraphPad Prism 9 (GraphPad Software, La Jolla, CA). A value of *p* ≤ 0.05 was considered significant.

## Results

### HFD-induced obesity results in severe EAE

**Figure 1:**
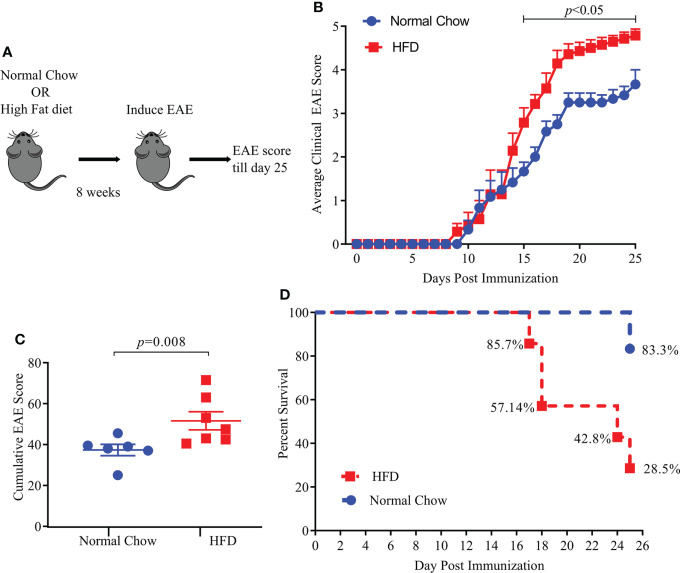
High fat diet (HFD) increases EAE severity in HLA-DR3 transgenic mice. **(A)** Model diagram representing day of diet initiation, EAE induction and disease scoring in HLA-DR3 transgenic mice. 6-8 weeks old HLA-DR3 transgenic mice were kept on Normal Chow (n= 6) and HFD (n= 7) for eight weeks. Mice were immunized with PLP_91-110_/CFA and pertussis toxin at days 0 and 2 to induce EAE. Mice were monitored for EAE disease progression before euthanizing on day 25 post-immunization. **(B)** Average clinical EAE scores from mice in A, acquired over time. **(C)** Cumulative EAE scores from mice in A. **(D)** Percent survival of mice in **(A)**, Data are representative of 3 independent experiments with 3-5 mice per group. Error bars represent the standard error of the mean for each experimental group. *p*-value was determined by 2-way ANOVA Sidak multiple comparisons test **(B)**, and Mann-Whitney test between the experimental groups **(C)**.

We used a standard HFD induced obesity model in HLA-DR3 transgenic mice which had been validated as an animal model of MS (18, 26). Six to eight-week-old HLA-DR3 transgenic mice were kept on a standard HFD diet or normal chow (NC) diet for eight weeks before induction of EAE (**Figure 1A**). HLA-DR3 transgenic mice kept on a HFD showed significant weight gain (**Supplementary Figure S1**) compared to mice on NC diet (NC vs HFD; 29.04 gm ± 3.60 vs 37.2 gm ± 3.3, *p* < 0.02) validating the use of HLA-DR3 transgenic mice for studying HFD induced obesity. As shown in **Figure 1B**, HLA-DR3 transgenic mice on a HFD diet showed higher average daily clinical score compared to those fed with NC diet. Additionally, HLA-DR3 fed with HFD showed a higher cumulative EAE score (HFD *vs*. NC; 51.57 ± 11.72 *vs.* 37.33 ± 6.73, *p* =0.008) plus increased mortality compared to mice on NC diet (**Figures 1C, D**). Disease onset was similar in mice fed with HFD or NC. These findings were replicated in a separate EAE model: C57BL/6 mice were immunized with MOG_35-55_/CFA (**Supplementary Figures S2A–D**). These results indicate that HFD induced obesity in HLA-DR3 mice resulted in the development of severe EAE compared to mice on NC.

### Depleting the gut microbiota in HFD-induced obese HLA-DR3 transgenic mice C57BL/6J mice decreases EAE severity

**Figure 2:**
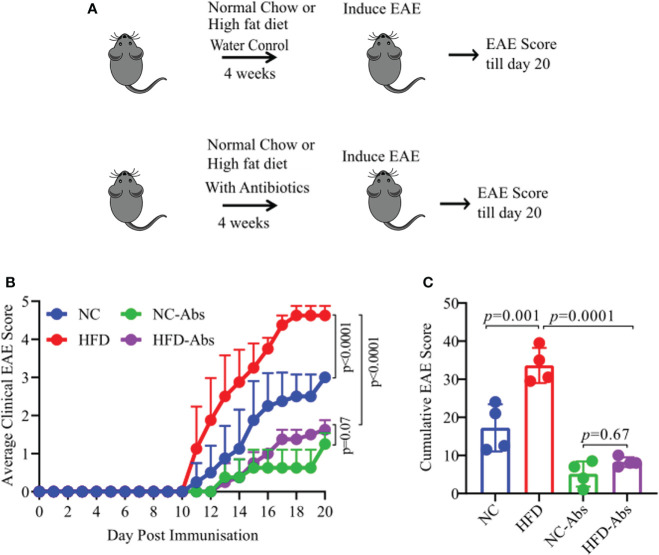
Gut bacteria depletion in HFD fed HLA-DR3 transgenic mice ameliorate EAE. **(A)** Model diagram representing day of diet initiation, antibiotics treatment, EAE induction and disease scoring in HLA-DR3 mice. 6-8-week-old male HLA-DR3 transgenic mice were placed on a HFD or a normal chow diet for 4 weeks while receiving broad spectrum antibiotics in the drinking water followed by immunization with PLP_91-110_/CFA to induce EAE. **(B)** Comparison of mean clinical scores in mice fed a HFD or normal chow diet and while receiving broad spectrum antibiotics in the drinking water. **(C)** Cumulative EAE scores from mice as in **(A)**. Data are representative of 4 mice per group. Error bars represent the standard error of the mean for each experimental group. *p*-value was determined by 2way ANOVA test **(B, C)**.

As diet have strongest influence on the composition of host gut microbiota which in turn can regulate host physiology (14, 15), we hypothesize that HFD induced obesity caused severe disease through modulation of gut microbiota. To test our hypothesis, we depleted gut microbiota of HLA-DR3 transgenic mice on a HFD or NC diet using broad spectrum antibiotic cocktail in drinking water for four weeks. Mice in control group were kept on HFD or NC diet and supplemented with water till induction of EAE. After four weeks on sterile or antibiotic water, EAE was induced in the above four groups of mice (HFD plus antibiotics, NC plus antibiotics, HFD plus water, and NC plus water) (**Figure 2A**). Depletion of gut bacteria in HFD induced obese mice exhibited significant amelioration of EAE compared to gut microbiota sufficient mice on a HFD or NC diet (**Figures 2B, C**). Similarly, mice on a NC diet and treated with Abx showed milder disease compared to untreated mice on a NC diet. These findings were replicated in a separate EAE model: C57BL/6 mice were immunized with MOG_35-55_/CFA (**Supplementary Figures S3A–C**). Thus, reduction of severe disease in HFD mice lacking gut bacteria indicate a critical role of gut microbiota in the development of severe disease in HLA-DR3 transgenic mice.

### HFD-induced obesity alters the gut microbiota

**Figure 3:**
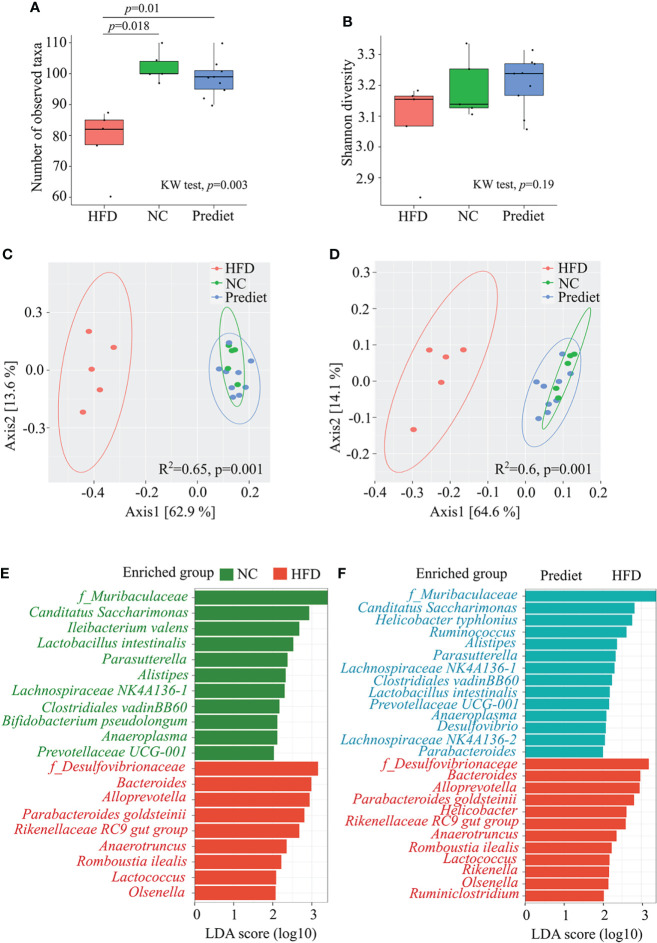
HFD induced obesity causes change in gut microbiota in HLA-DR3 transgenic mice. Amplicon based microbial diversity of mice on a prediet (n=9), a high fat diet (HFD, n=5) or normal chow (NC, n=5). Alpha diversity measures obtained using **(A)** Observed taxa, **(B)** Shannon diversity. PCOA plot representing beta diversity index measured using **(C)** Bray-curtis dissimilarity metrics, **(D)** Weighted unifrac measures among the groups. **(E)** Bar plot representing significantly altered taxa between HFD and NC, and **(F)** HFD and prediet conditions following lefse analysis in R among all taxonomic ranks with *lda_cutoff* of 2 and *wilcoxon_cutoff* of 0.01, and *kw_cutoff* of 0.01 respectively. Kruskal wallis (KW) test was performed for **(A–C)** followed by pairwise comparison using wilcox test and *p*-value with Benjamini Hochberg (BH) correction. *adonis* test was performed for **(D, E)**.

As depletion of gut microbiota resulted in loss of severe EAE in obese mice, next we investigated a direct effect of HFD-induced obesity on gut microbiota composition to identify the specific bacteria linked with disease modulation. We kept HLA-DR3 transgenic mice on HFD or NC for 8 weeks and performed 16S ribosomal RNA (rRNA) (V3-V4) sequencing of bacterial DNA isolated from their feces. We observed that the total number of observed ASVs (amplicon sequence variants) were significantly reduced in mice kept on HFD compared to mice on NC diet (*p*=0.018) and prediet (*p*=0.01) (**Figure 3A**). At the same time, no significant change was observed between prediet and NC conditions (*p*=0.22) (**Figure 3A**). Similar trends were observed with Shannon diversity (**Figure 3B**). The analysis of beta diversity indicated that the microbial communities formed in the context of a HFD were significantly different compared to those from mice fed NC or from pre-diet samples (**Figures 3C, D**). When comparing the NC with HFD, the relative abundances of *Proteobacteria* was increased while *Patescibacteria* was decreased at phylum level (**Supplementary Figure S4A**). Similarly, at family level, *Desulfovibrionacaeae*, *Bacteroidaceae*, *Lachnospiraceae* and *Tanerellaceae* were increased while *Muribaculaceae* was reduced (**Supplementary Figure S4B**). Upon *lefse* analysis, taxa belonging to *Desulfovibrionaceae* family and *Proteobacteria* phylum along with *Bacteroides* genus were significantly enhanced in mice on a HFD when compared to mice on a NC (**Figure 3E**). Gut microbiota changes between the NC diet and prediet conditions were smaller compared to gut microbiota changes observed in HFD group. Microbial taxa belonging to *Muribaculaceae* and *Lachnospiraceae* were significantly increased in NC diet and similar microbial enrichments were observed in the prediet condition compared with HFD group (**Figure 3F**, **Supplementary Figure S4B**), suggesting that changes in HFD group is not age associated but due to HFD. Thus, our findings indicate that disease severity in HFD-induced obese mouse was associated with alteration of gut microbiota with an increased abundance of *Proteobacteria* phylum, and *Desulfovibrionaceae* family bacteria.

### HFD induced obesity increases abundance of key biosynthesis pathways associated with gut inflammation

**Figure 4:**
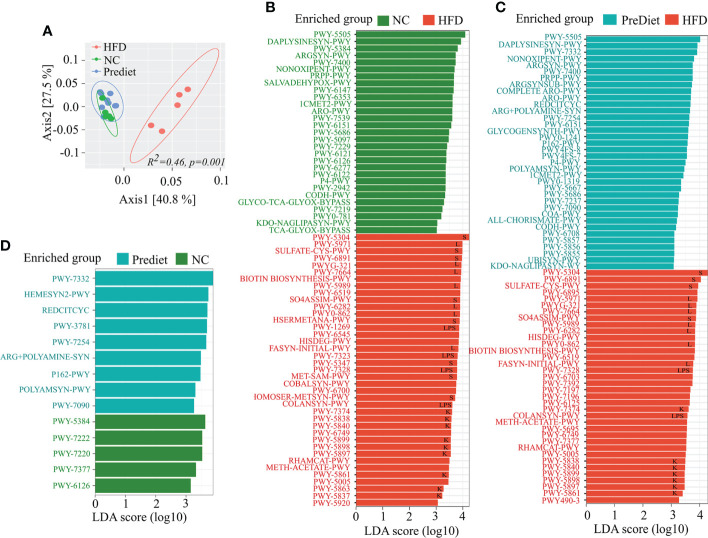
Functional investigation of the microbial communities in HLA-DR3 Transgenic Mice Fed with HFD and NC. **(A)** PCoA representing differentially clustering patterns of HFD, NC and prediet communities using Bray Curtis dissimilarity metrics. Lefse analysis showing functional pathways significantly enriched in **(B)** HFD compared to NC, **(C)** HFD compared to prediet conditions and, **(D)** NC and prediet conditions. Lefse analysis was carried out with *lda_cutoff* of 3 and *wilcoxon_cutoff* of 0.01, and *kw_cutoff* of 0.01 respectively with other parameters remaining default. Functional pathways related to sulfur metabolism, long chain fatty acid synthesis, lipopolysaccharides and vitamin K are represented by “S”, “L”, “LPS” and “K” respectively in **(B–D)**.

To determine the functional profile of the microbiota from the HFD and NC groups, we performed functional analysis using PICRUSt2 (18) and the MetaCyc database. Functionally, the HFD and NC diet or prediet microbial communities were distinctly separated (*p*=0.001) from one another (**Figure 4A**) indicating major functional changes in the HFD group compared to NC or prediet conditions. Further comparison of pathways between NC and HFD exhibited significant enrichment of sulfur metabolism, long chain fatty acid biosynthesis, lipopolysaccharide biosynthesis, and Vitamin K metabolism in HFD group (**Figure 4B**, **Supplementary Figure S5**). Identical enrichment of the metabolic pathways was observed in HFD when compared to the prediet conditions (**Figure 4C**, **Supplementary Figure S5**). Interestingly, no such changes were observed between the NC diet and prediet conditions (**Figure 4D**, **Supplementary Figure S5**). Altogether, functional analysis of microbiome data shows a significant alteration of the metabolic pathways in HFD compared to NC or prediet conditions which suggest that HFD induced alteration of gut microbial metabolic pathway might have contributed to the severity of the EAE in HFD fed mice.

### HFD- induced obesity increases gut permeability

**Figure 5:**
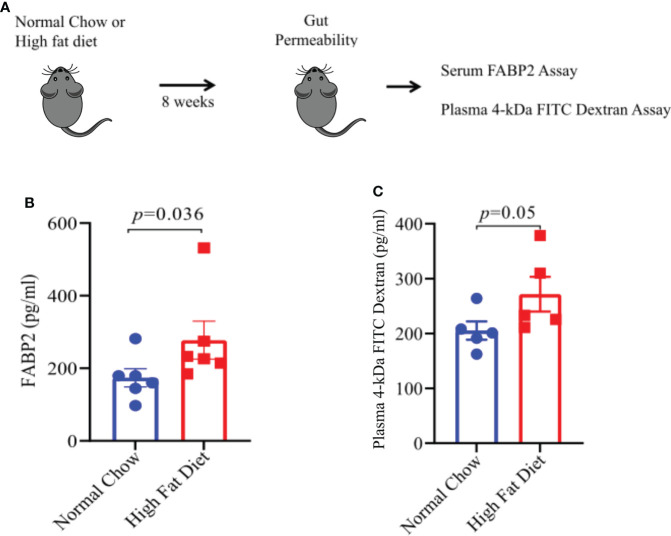
A High Fat Diet results in increase gut permeability. **(A)** Schematic of experimental design. 6-8-week old male mice were placed on the indicated diet for 8 weeks prior to FITC-dextran permeability assay and FABP2 assay. For FITC-dextran permeability assay, mice on either diet were fasted for 6 hours prior to oral treatment with 4 kDa FITC-dextran. Serum samples were taken 2 hours post-treatment. Sera level of FITC was measured, using a spectrophotometer, as a measurement of intestinal permeability. For FABP2 assay, blood sera samples were analyzed by ELISA as per manufacturer protocol. **(B)** FABP2 concentrations in HFD and Normal chow mice. **(C)** 4 kDa FITC-dextran level in HFD and Normal chow mice. Error bars represent the standard error of the mean for each experimental group. *p-*value was determined by Mann-Whitney test between the experimental groups in **(B, C)**.

One of the mechanisms through which gut dysbiosis can cause inflammation is by increasing the gut permeability that leads to leakage of bacterial products and inflammatory mediator markers into systemic circulation. Increased gut permeability and high serum levels of intestinal-fatty acid binding proteins (FABP2) are a potential biomarker of compromised intestinal barrier (27). We argued that HFD induced obesity and associated gut microbiota alteration might cause intestinal damage leading to increased gut permeability and FABP2 expression levels in systemic circulation. To test the same, we kept mice on HFD or NC diet for 8 weeks (**Figure 5A**) and measured the plasma levels of FABP2 in both groups. Plasma FABP2 levels were higher in mice kept on HFD compared to mice on NC diet (**Figure 5B**). To further confirm that HFD-induced obesity increases gut permeability, we performed an FITC-dextran intestinal permeability assay which is a surrogate of leaky gut (**Figure 5C**). The mice on HFD showed higher FITC- absorbance in sera at 2 hours after gavage with FITC-dextran suggesting an increase in intestinal permeability in mice on HFD compared to mice on a NC diet (**Figure 5C**). Thus, our findings suggest that alterations in the gut microbiota composition and function can induce gut permeability with increased intestinal markers in systemic circulation in HFD fed mice. This can induce pro-inflammatory mediators which may be involved in EAE severity.

### HFD- induced obesity in HLA-DR3 transgenic mice increases pro-inflammatory cytokine levels

**Figure 6:**
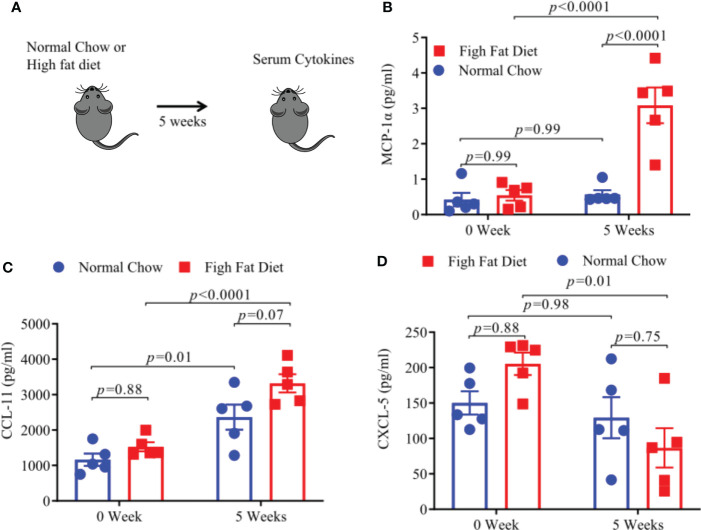
A High Fat Diet results in increase pro-inflammatory cytokines. **(A)** Schematic of experimental design. 6-8-week old HLA-DR3 transgenic male mice were placed on the indicated diet and after 5 weeks sera were collected from blood. MCP-1a concentrations post diet **(B)**, CCL-11 concentrations post diet **(C)**, CXCL-5 concentrations post diet **(D)**. Error bars represent the standard error of the mean for each experimental group. *p*-value was determined by 2way ANOVA Sidak multiple comparisons test for **(B–D)**.

Increased levels of pro-inflammatory cytokines and chemokines, associated with a low-grade inflammatory state, have been found in obese individuals (28–30). We measured levels of pro-inflammatory cytokines and chemokines using a Bioplex assay after keeping HLA-DR3 transgenic mice on HFD or NC diet for 5 weeks (**Figure 6A**). We observed that levels of MCP1-α and CCL-11 were significantly increased in mice fed with HFD (**Figures 6B, C**) whereas, CXCL5 levels were significantly decreased in HFD mice compared to mice on a NC diet. (**Figure 6D**). Thus, mice on HFD have increased levels of MCP1-α and CCL-11 chemokine compared to mice on NC diet which point towards their potential role in modulating EAE severity.

## Discussion

In the last few years epidemiological studies have shown that obesity increases the risk and severity (frequent disease relapse) of MS (10, 31). Although gut dysbiosis (altered microbial flora compared to healthy control) emerged as crucial environmental factor in both obesity (14, 15) and MS (6, 7, 10), the importance of obesity on the gut microbiome and its implications on the severity of MS is unknown. In the present study, utilizing HLA-DR3 transgenic mouse model of MS, we showed that HFD- induced obesity increased EAE severity through modulation of gut microbiota. Specifically, mice on HFD showed an enrichment of *Proteobacteria* and *Desulfovibrionaceae* bacteria. Additionally, functional analysis showed an increase in sulfur metabolism, lipopolysaccharide, and long chain fatty acid bacterial pathways. Finally, HFD mice also showed feature of leaky gut characterized by increased gut permeability, translocation of intestinal protein and high levels of pro-inflammatory mediators in systemic circulation. Collectively, our results suggest that obesity causes severe disease in the animal model of MS through alteration of gut microbiota and metabolic pathways resulting in leaky gut and systemic pro-inflammatory immune response.

Our data on induction of severe EAE in HFD mice indicate that environmental factors specifically diet and gut microbiome have strong influence on autoimmune disease such as MS. Previous studies have also shown that HFD induces severe EAE in C57BL/6J mice (32). These studies reported that HFD causes an increase in pro-inflammatory cytokines such as TNF-α, IL-6 and CCL-2 that can expand pro-inflammatory Th17 subset which can induce severe EAE. However, these studies did not provide mechanisms through which HFD can cause an increase in pro-inflammatory response. Here we provide the missing link between HFD induced obesity and severe EAE disease. Our data indicate that gut microbiome and associated metabolic pathways induced in mice on HFD is one of the mechanisms through which obesity can modulate EAE. Loss of disease severity in mice depleted of gut bacteria (Abs Rx) further strengthen our hypothesis that HFD-induced changes in gut microbiota are responsible for HFD mediated pathogenic response.

HFD mice showed significantly reduced alpha diversity in comparison to normal chow and pre diet mice as seen previously (33). Microbial diversity is a critical parameter as diversity directly affects the ability of the microbiome to resist pathogenic bacterial invasion (34). Furthermore, excess nutrient loading can cause decreased microbial diversity as a small number of species can overgrow and outcompete other species (34). The consequences of low bacterial diversity are highlighted in human diseases. For example, studies have shown that patients with inflammatory bowel disease (IBD) have lower bacterial diversity in comparison to healthy controls (35). HFD induced obese mice may be particularly susceptible to pathogen invasion due to reduced alpha diversity leading to a pro-inflammatory state thereby exacerbating EAE. Our study provides an unique insight into gut microbiota differences between HFD and NC, in the context of EAE, opening the door for further studies characterizing the impact of HFD induced gut bacteria on EAE pathogenesis.

Beside bacterial diversity, HFD mice have a distinct microbiota composition in comparison to standard normal chow diet. The alterations in microbiota composition are highlighted by an increase in *Proteobacteria*, and *Desulfovibrionaceae Proteobacteria* have been associated with a pro-inflammatory state and have been shown to be significantly increased in Ulcerative colitis (UC) patients (36). *Proteobacteria* have also been shown to be positively correlated with UC severity (36). Notably, transfer of *Proteobacteria* species were sufficient to provoke UC in mice without any genetic immune defects (37). Additionally, TLR5 deficient mice spontaneously develop colitis and exhibit dysbiosis characterized by an outgrowth of *Proteobacteria.* These data indicate a pro-inflammatory role of *Proteobacteria* in the gut. Therefore, enhanced growth of *Proteobacteria* in HFD mice may exacerbate gut inflammation leading to systemic inflammation and CNS autoimmunity. Further, within *Proteobacteria*, we specifically observed the increase in members of *Desulfovibrionaceae* family in mice kept on HFD. The increase in *Desulfovibrionaceae* is common in adult and pediatric MS patients (8, 38). *Desulfovibrionaceae* includes genera of sulfate-reducing bacteria that produce higher level of hydrogen sulfide (H_2_S) as a byproduct in the gut. Interestingly, we also observed an enrichment of bacterial H_2_S metabolic pathways. Combined together, our data suggest that HFD induced gut microbiota alteration, specifically enrichment of H_2_S producing bacteria (*Desulfovibrionaceae*) and H_2_S pathways might be involved in induction of pro-inflammatory response leading to severe EAE disease. Importance of H_2_S pathways in modulating inflammatory pathways had been showed earlier (39) such as elevated level of sulfate- reducing bacteria in feces of patients with UC and IBD (40). A higher concentration of H_2_S were also reported to be genotoxic and pro-inflammatory as it can increase the production of proinflammatory cytokines such as IL-1β and IL-6 from IECs (39). Thus, HFD enriched pathogenic bacteria can increase H_2_S which then can induce IECs for production of IL-1β plus IL-6 and promote expansion of pro-inflammatory Th17 cells resulting in induction of sever MS/EAE.

Additionally, functional pathways associated with long chain fatty acid synthesis and lipopolysaccharides were more abundant in HFD diet mice (**Figure 4**). The increased long chain fatty acids and lipopolysaccharide pathways have also been previously shown to associate with gut inflammation (41). Furthermore, increased abundance of bacterial lipopolysaccharide plays an important role in gut inflammation (42). Higher lipopolysaccharide biosynthesis in HFD mice can cause defective intestinal tight junction and increases intestinal permeability and systemic inflammation (42). High serum levels of intestinal-fatty acid binding proteins (FABP2) are a potential biomarker of intestinal barrier dysfunction and increased gut permeability (27). Role of FABP2 in intestinal barrier dysfunction in the case of obesity induced EAE severity is not known. An increased level of FABP2 has been reported in chronically elevated glucose levels in obese individuals (43). Our findings showed that serum FABP2 level were higher in mice on HFD compared to mice on NC diet mice. Further analysis of gut permeability using FITC-dextran intestinal permeability assay which is a surrogate of leaky gut confirmed that HFD-induced obesity increased intestinal permeability in mice on HFD compared to mice on NC diets. Maintaining gut barrier integrity is critical to physiological homeostasis as trillions of bacteria present in the gut can stimulate a pro-inflammatory immune response if allowed access to gut associated lymphoid tissue (44). The increase in the gut permeability by HFD has also been described previously (29). Previous studies in MS patients have also shown an increase in gut permeability (45). Thus, HFD induced gut dysbiosis and resulting increase in gut permeability can modulate disease severity through translocation of pro-inflammatory bacteria/bacterial products in systemic circulation and induction of pro-inflammation state. We found an increase in pro-inflammatory chemokines and cytokines such as MCP-1α, CCL-11, and decrease in CXCL-5 level in HFD fed mice, compare to NC fed mice. MCP-1 had been shown to induce Th1 immune responses during EAE and promote macrophage recruitment to the inflamed CNS (46). This phenomenon is essential for primming of T cells to execute a Th1 effector program in EAE (46). CCL-11 had been evaluated for their potent role in immunomodulation and as a biomarker of human disease (47). Elevated plasma levels of MCP-1 and CCL-11 have been also reported in neurodegenerative diseases (48) suggesting that HFD induced obesity can trigger EAE severity though induction of proinflammatory MCP-1 and chemokines such as CCL-11. Our findings on low sera of CXCL-5 in sera of HFD mice is in contrast to prior study showing higher levels of CXCL-5 in mice with EAE and cerebrospinal fluid of MS patients during relapse compared with remission phase of disease (49). However, the role of CXCL-5 in chronic inflammatory diseases is not clearly understood. As circulating CXCL-5 levels had been inversely correlated with atherosclerosis severity, also suggesting a possible protective role for CXCL-5 (50). However, it remains to be established whether these levels correlate with the disease progression and EAE severity in HFD induced obese mice.

There are some limitations to our study as we didn’t profile gut microbiome, metabolites, or systemic inflammatory mediators post-EAE. The rationale for the same is that induction of EAE itself results in modulation of gut microbiome, metabolome, gut permeability, and/or systemic inflammatory mediators. Therefore, it is difficult to differentiate the effects due to obese EAE vs normal chow EAE. e.g. Prior studies have shown that EAE induction causes gut dysbiosis with decreases in gut commensal bacteria such as *Lactobacillaceae* (51). Interestingly, in the present study, we have observed a decreasing trend in *Lactobacillaceae* in mice fed with HFD compared to mice fed with NC (**Supplementary Figure S4**). Similarly EAE induction itself increases gut permeability at both onset (day 7) and peak of EAE (day 14) (52) and thus measuring gut permeability post-EAE might not help in differentiating gut permeability effect due to obesity from those due to disease itself. Additionally, majority of systemic inflammatory mediators modulated in mice on HFD are also modulated in EAE itself. However, it is possible that there are subtle changes in gut microbiome, metabolome and/or systemic inflammatory mediators post-EAE between mice on HFD vs NC and future studies are warranted to determine the same.

In summary, we show that HFD induced obesity leads to increased EAE severity through modulation of gut bacteria especially enrichment of pathogenic bacteria linked with sulfate reduction and lipopolysaccharide biosynthesis pathways, which then can induce systemic inflammation through induction of gut permeability and pro-inflammatory mediators. Future studies dissecting the role of specific bacterial metabolite such as H_2_S and long chain fatty acids in modulating EAE disease will help in defining the precise mechanism through which HFD induced gut bacteria can influence the pathobiology of MS.

## Data availability statement

The gut bacterial sequence data presented in the study are deposited in the NCBI repository, accession number PRJNA842401. The data is available here: https://www.ncbi.nlm.nih.gov/bioproject/PRJNA842401.

## Ethics statement

Mice were bred and maintained in the University of Iowa animal facility in accordance with NIH and institutional guidelines. All experiments were approved by the Institutional Animal Care and Use Committee at the University of Iowa.

## Author contributions

SS conceptualized the study, designed, and performed the experiments, and wrote the manuscript and gave final approval of the manuscript to be published. AM conceptualized, designed the study, edited the manuscript, and gave final approval of the manuscript to be published. SG performed data analysis and helped in writing manuscript. PL helped with performing experiments. All authors contributed to the article and approved the submitted version.

## Funding

The Author acknowledge funding from the National Multiple Sclerosis Society grant (RG 5138A1/1T), National Institutes of Health/NIAID (1R01AI137075), the University of Iowa Environmental Health Sciences Research Center, NIEHS/NIH (P30 ES005605), and a gift from P. Heppelmann and M. Wacek to AM.

## Acknowledgments

We thank the Drs. Karandikar, Jabbari, and Lieberman laboratories for helpful discussion.

## Conflict of interest

Author AM is inventor of a technology claiming the use of *Prevotella histicola* for the treatment of autoimmune diseases. The patent for the technology is owned by Mayo Clinic, who has given exclusive license to Evelo Biosciences. AM received royalties from Mayo Clinic (paid by Evelo Biosciences). However, no fund or product from the patent were used in the present study.

The remaining authors declare that the research was conducted in the absence of any commercial or financial relationships that could be construed as a potential conflict of interest.

## Publisher’s note

All claims expressed in this article are solely those of the authors and do not necessarily represent those of their affiliated organizations, or those of the publisher, the editors and the reviewers. Any product that may be evaluated in this article, or claim that may be made by its manufacturer, is not guaranteed or endorsed by the publisher.
