# Supplementary material for: Obesity induced gut dysbiosis contributes to disease severity in an animal model of multiple sclerosis
Source: Front Immunol. 2022 Sep 9;13:966417. doi: 10.3389/fimmu.2022.966417 (PMC9509138; doi:10.3389/fimmu.2022.966417)
Supplement: Supplementary file 1 [file Presentation_1.pptx]

## Slide 1
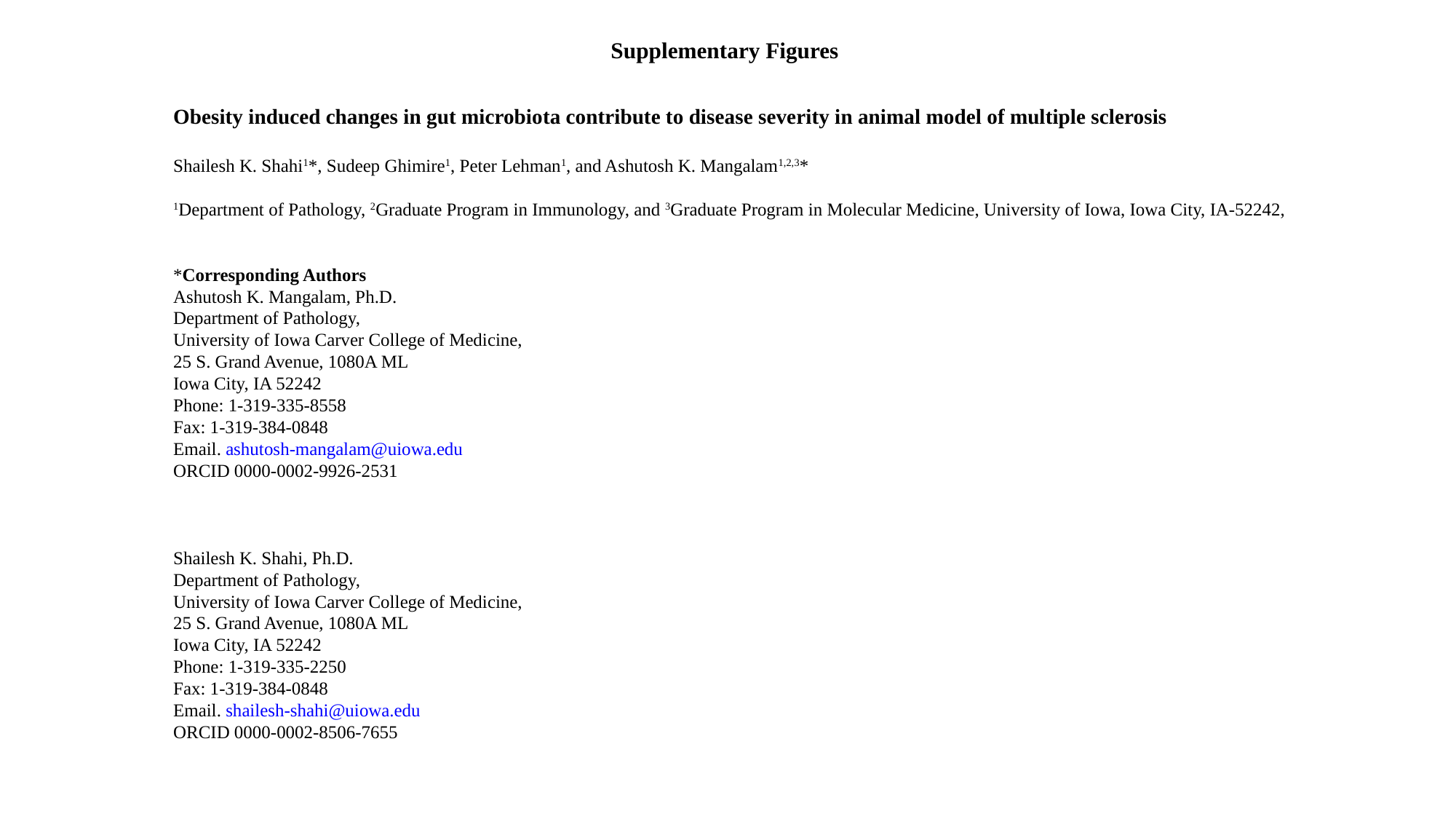

Supplementary Figures
Obesity induced changes in gut microbiota contribute to disease severity in animal model of multiple sclerosis
Shailesh K. Shahi1*, Sudeep Ghimire1, Peter Lehman1, and Ashutosh K. Mangalam1,2,3*
1Department of Pathology, 2Graduate Program in Immunology, and 3Graduate Program in Molecular Medicine, University of Iowa, Iowa City, IA-52242,
*Corresponding Authors
Ashutosh K. Mangalam, Ph.D.
Department of Pathology,
University of Iowa Carver College of Medicine,
25 S. Grand Avenue, 1080A ML
Iowa City, IA 52242
Phone: 1-319-335-8558
Fax: 1-319-384-0848
Email. ashutosh-mangalam@uiowa.edu
ORCID 0000-0002-9926-2531
Shailesh K. Shahi, Ph.D.
Department of Pathology,
University of Iowa Carver College of Medicine,
25 S. Grand Avenue, 1080A ML
Iowa City, IA 52242
Phone: 1-319-335-2250
Fax: 1-319-384-0848
Email. shailesh-shahi@uiowa.edu
ORCID 0000-0002-8506-7655

## Slide 2
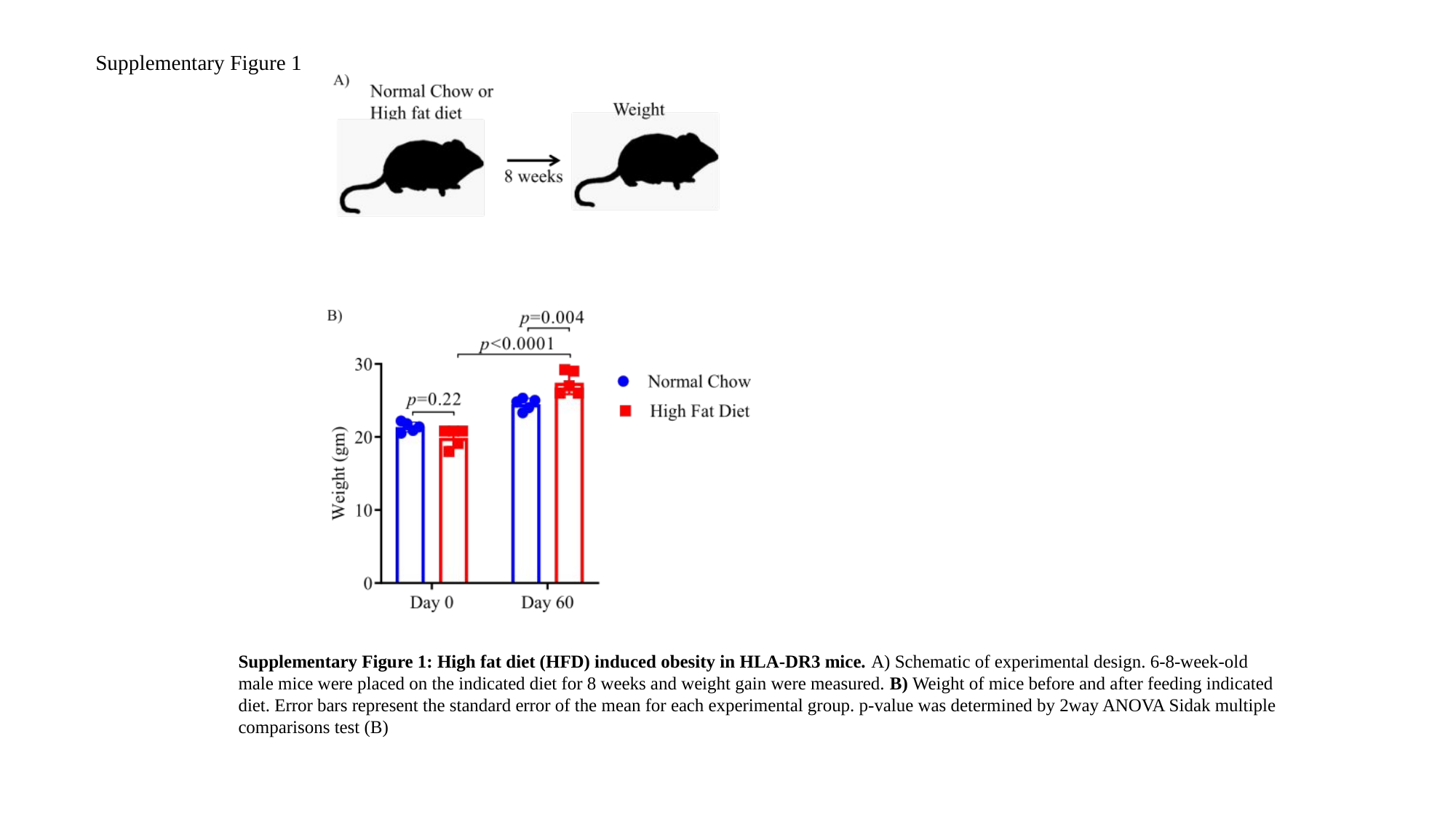

Supplementary Figure 1
Supplementary Figure 1: High fat diet (HFD) induced obesity in HLA-DR3 mice. A) Schematic of experimental design. 6-8-week-old male mice were placed on the indicated diet for 8 weeks and weight gain were measured. B) Weight of mice before and after feeding indicated diet. Error bars represent the standard error of the mean for each experimental group. p-value was determined by 2way ANOVA Sidak multiple comparisons test (B)

## Slide 3
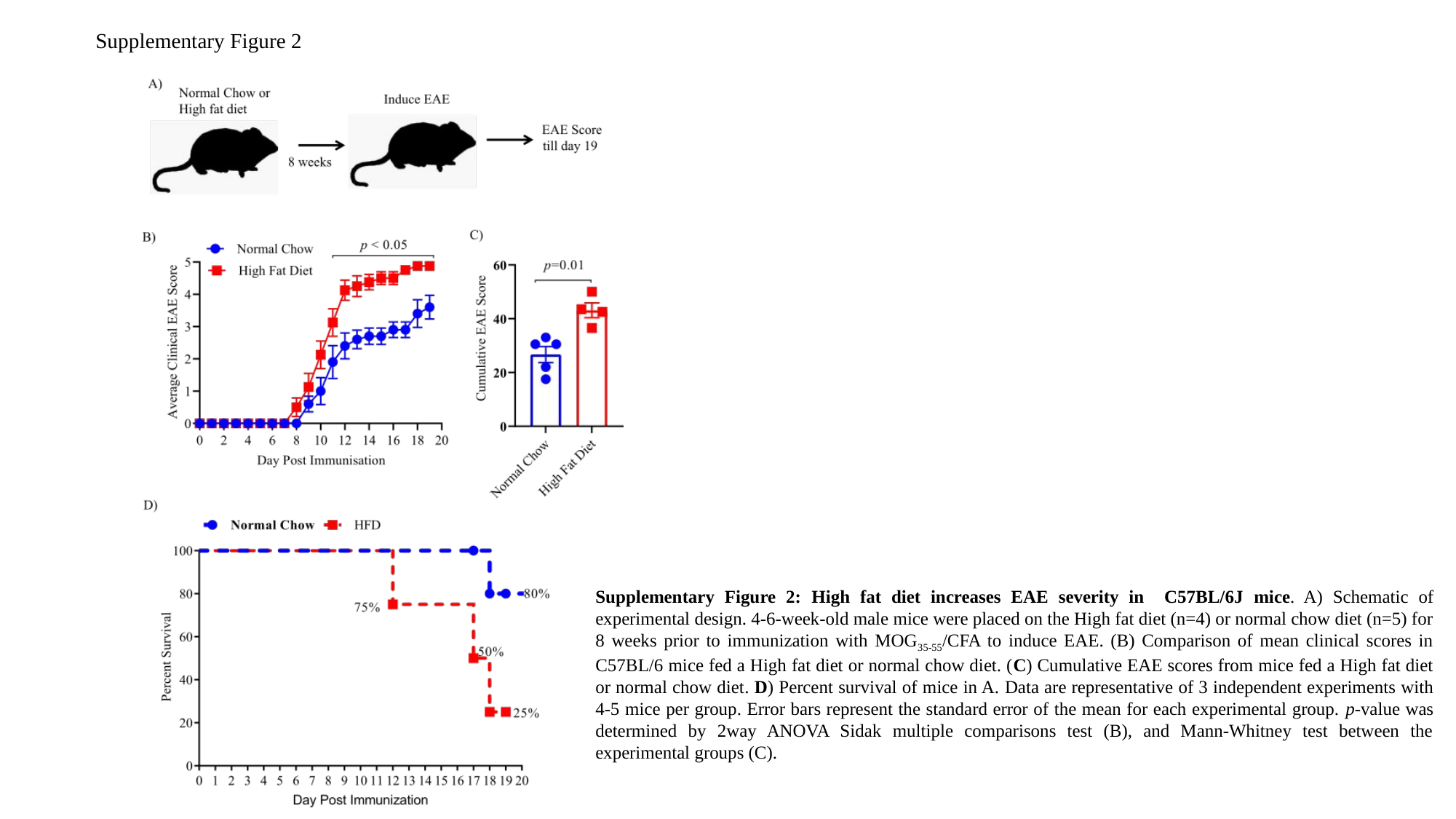

Supplementary Figure 2
Supplementary Figure 2: High fat diet increases EAE severity in C57BL/6J mice. A) Schematic of experimental design. 4-6-week-old male mice were placed on the High fat diet (n=4) or normal chow diet (n=5) for 8 weeks prior to immunization with MOG35-55/CFA to induce EAE. (B) Comparison of mean clinical scores in C57BL/6 mice fed a High fat diet or normal chow diet. (C) Cumulative EAE scores from mice fed a High fat diet or normal chow diet. D) Percent survival of mice in A. Data are representative of 3 independent experiments with 4-5 mice per group. Error bars represent the standard error of the mean for each experimental group. p-value was determined by 2way ANOVA Sidak multiple comparisons test (B), and Mann-Whitney test between the experimental groups (C).

## Slide 4
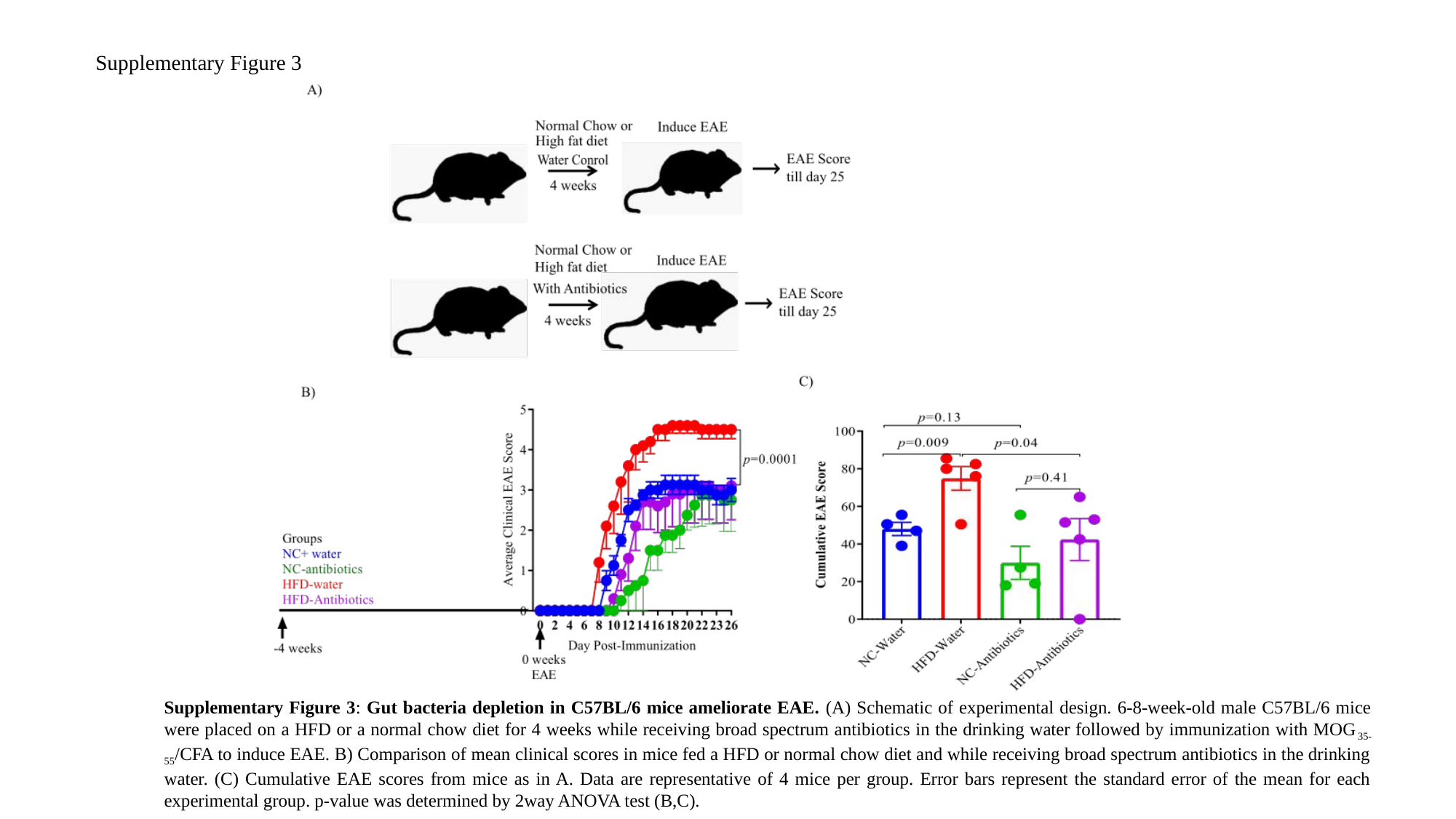

Supplementary Figure 3
Supplementary Figure 3: Gut bacteria depletion in C57BL/6 mice ameliorate EAE. (A) Schematic of experimental design. 6-8-week-old male C57BL/6 mice were placed on a HFD or a normal chow diet for 4 weeks while receiving broad spectrum antibiotics in the drinking water followed by immunization with MOG35-55/CFA to induce EAE. B) Comparison of mean clinical scores in mice fed a HFD or normal chow diet and while receiving broad spectrum antibiotics in the drinking water. (C) Cumulative EAE scores from mice as in A. Data are representative of 4 mice per group. Error bars represent the standard error of the mean for each experimental group. p-value was determined by 2way ANOVA test (B,C).

## Slide 5
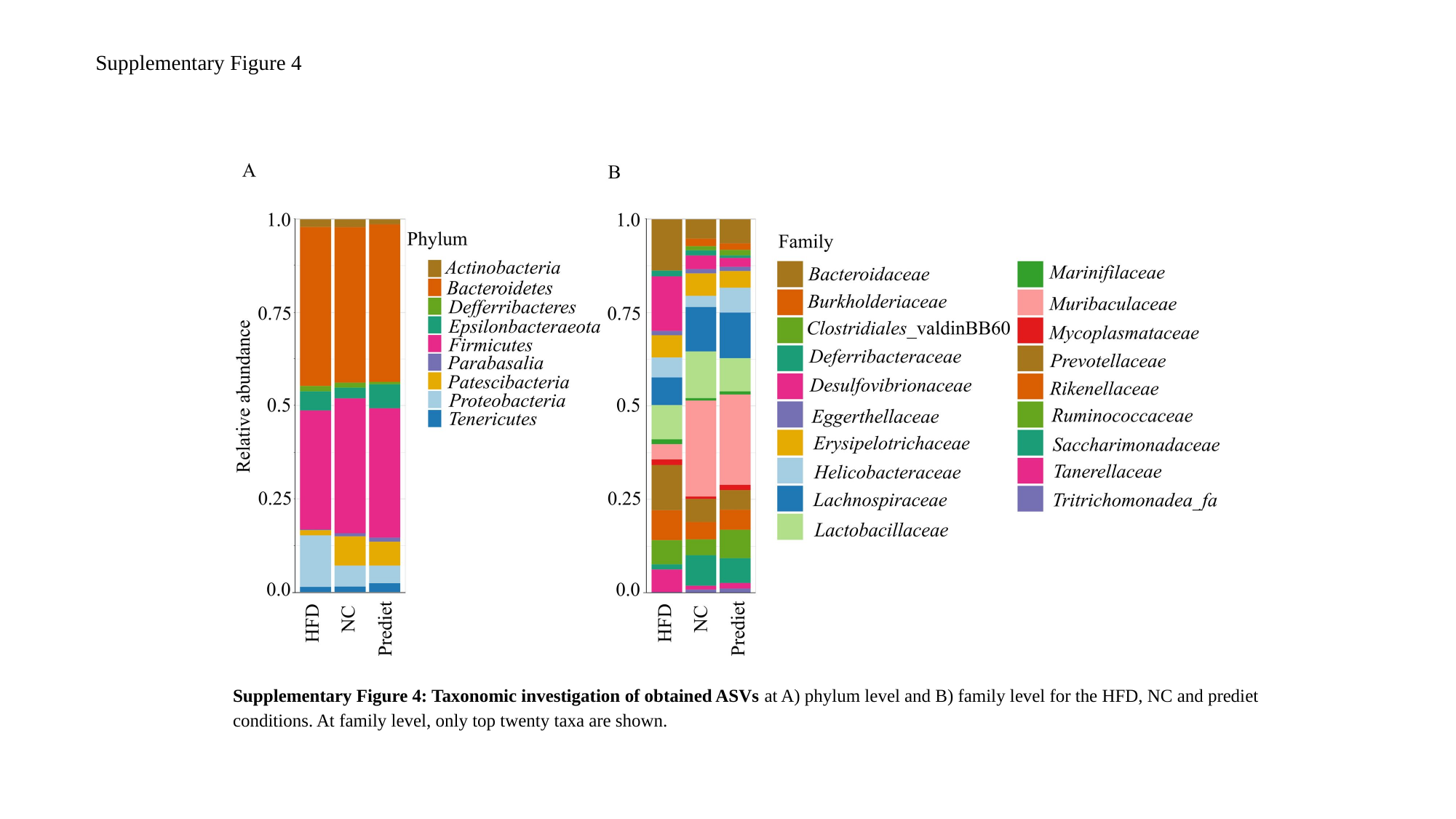

Supplementary Figure 4
Supplementary Figure 4: Taxonomic investigation of obtained ASVs at A) phylum level and B) family level for the HFD, NC and prediet conditions. At family level, only top twenty taxa are shown.

## Slide 6
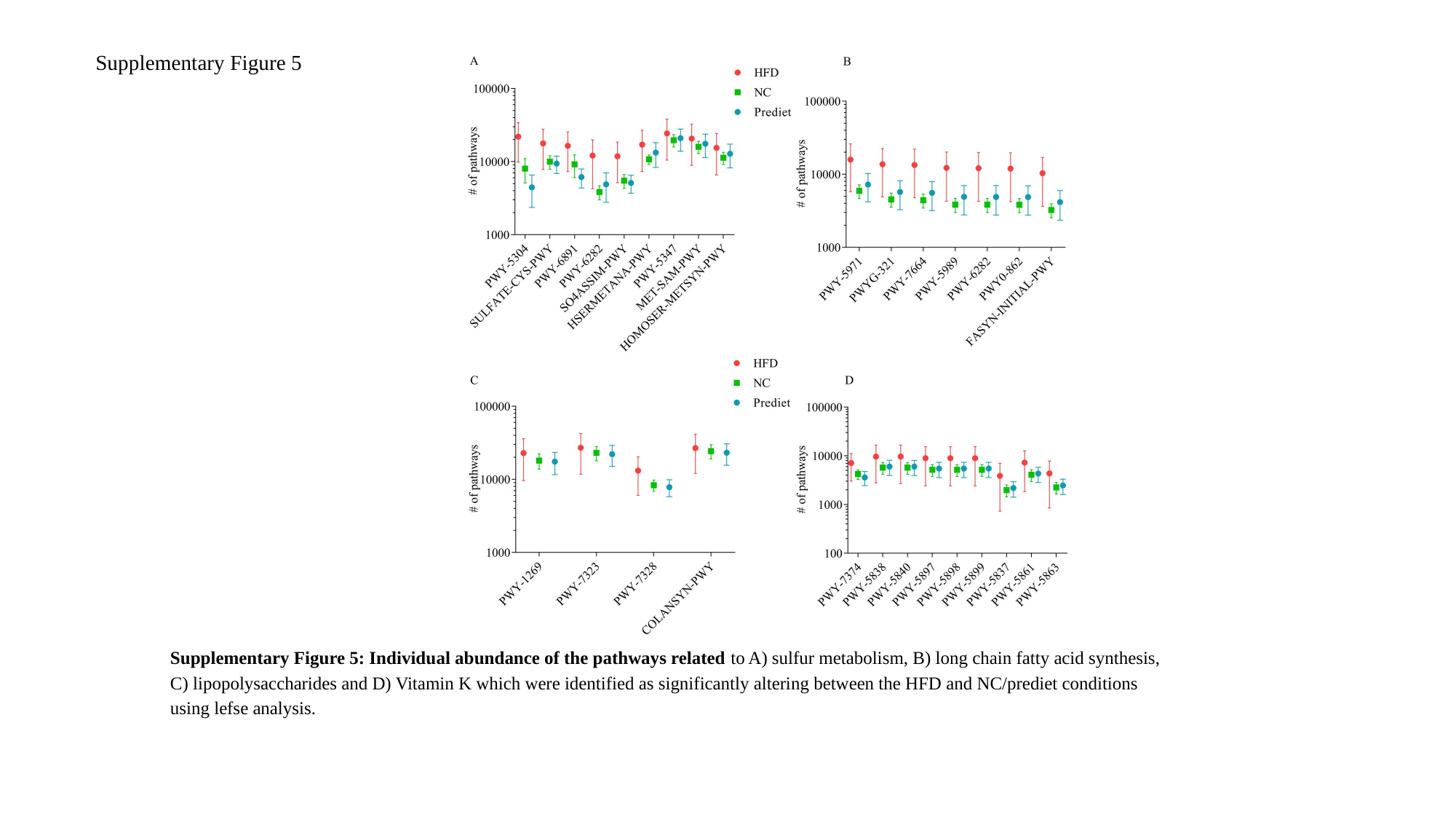

Supplementary Figure 5
Supplementary Figure 5: Individual abundance of the pathways related to A) sulfur metabolism, B) long chain fatty acid synthesis, C) lipopolysaccharides and D) Vitamin K which were identified as significantly altering between the HFD and NC/prediet conditions using lefse analysis.
